# Evaluation of Treatment Outcomes Among Individuals on Highly Active Antiretroviral Therapy in KwaZulu-Natal, South Africa

**DOI:** 10.1155/arat/8834740

**Published:** 2024-12-10

**Authors:** Tambwe Willy Muzumbukilwa, Riziki Ghislain Manimani, Aganze Gloire-Aime Mushebenge, Rajesh Vikram Vagiri, Manimbulu Nlooto

**Affiliations:** ^1^Department of Pharmaceutical Sciences, Discipline of Pharmaceutical Sciences, School of Health Sciences, Westville Campus, University of KwaZulu-Natal, University Road, Durban 4001, South Africa; ^2^Department of Internal Medicine, Nelson R. Mandela School of Medicine, University of KwaZulu-Natal, Durban 4001, South Africa; ^3^Department of Health, Center of Research in Natural Science Lwiro, Bukavu, Democratic Republic of the Congo; ^4^Department of Pharmacy, School of Health Care Sciences, Faculty of Health Sciences, University of Limpopo, Limpopo, South Africa

**Keywords:** HAART, HIV/AIDS, KwaZulu-Natal, PLWHA, treatment outcomes

## Abstract

Despite access to antiretroviral therapy (ART), South Africa has a high human immunodeficiency virus (HIV) burden. Treatment outcomes among individuals on highly active ART (HAART) in KwaZulu-Natal, with a higher incidence of HIV, are not fully known. This study evaluated the impact of HAART outcomes and identified and analyzed the factors associated with the outcomes in people living with HIV and AIDS (PLWHA) in the high-incidence region of KwaZulu-Natal Province, South Africa. This retrospective medical record review was conducted at King Edward VIII Hospital in South Africa. Data analysis was performed using STATA software Version 18.0 and Microsoft Excel 2021. The estimates used were 95% confidence intervals, and a *p* value < 0.05 was considered statistically significant. A total of 707 clinical records of PLWHA were examined and analyzed; less than half of them (44.98%, *n* = 318) achieved the benchmark of two consecutive instances of suppressed viral loads. The CD4 greater than or equal to 500 cells/mm^3^ at baseline average of 22.91% (*n* = 162) registered an increase to 48.94% (*n* = 346) in the 6^th^ month and further escalated to 79.49% (*n* = 562) by the 12^th^ month following ART initiation. A total of 160 deaths (mortality rate of 22.63%) were recorded within the study period. The percentage of HIV-infected patients attaining viral suppression at 6 and 12 months after initiating the treatment was respectively 44.98% and 67.04%, below the 90% target established by the Joint United Nations Program on HIV/AIDS (UNAIDS). The proportion of favorable immunological responses for individuals on ART increased over time.

## 1. Introduction

Despite substantial progress in the expansion and implementation of human immunodeficiency virus (HIV) treatment and prevention initiatives, HIV/AIDS remains a major public health challenge [1]. As evidenced by global data from 2021, 1.5 million newly reported HIV infections and 650,000 deaths were attributed to HIV- and AIDS-related illnesses [2]. According to academic research, East and Southern Africa bear a significant burden of the HIV pandemic, accounting for nearly half of new HIV infections and 40% of HIV- and AIDS-related deaths [3]. Within this region, South Africa stands out with its adult HIV prevalence reaching 20%, making it the country with the highest HIV epidemic worldwide [4]. Presently, approximately 7.5 million individuals in South Africa are living with HIV and AIDS. Notably, a substantial portion of this affected population, specifically 18.23%, resides in KwaZulu-Natal, making it the province with the highest number of HIV cases globally [5].

Throughout its evolution, South Africa's national antiretroviral therapy (ART) guidelines have progressively adjusted towards more inclusive initiation thresholds [6, 7]. In September 2016, the country adopted the universal test and treat (UTT) strategy, as recommended by the World Health Organization (WHO) [8]. This strategic shift resulted in the implementation of expanded guidelines that advocate for initiating ART in all individuals diagnosed with HIV, regardless of conventional clinical indicators and thresholds, such as CD4 cell counts and WHO clinical stage [8, 9].

Rapid initiation of highly active ART (HAART) has emerged as a crucial factor in enhancing ART access, facilitating sustained viral load (VL) suppression, restoring immune function, improving the quality of life, clinical staging according to WHO guidelines, preventing transmission, and reducing HIV- and AIDS-related morbidity and mortality [10–13]. In this regard, access to ART has witnessed a significant and rapid expansion, resulting in approximately 74% of individuals with HIV gaining access to ART in South Africa [14].

The South African National Department of Health (NDoH) has issued guidelines endorsing the use of a VL threshold of ≤ 50 copies/mL to define the state of viral suppression. In the context of viral failure, the NDoH defines it as having two consecutive VL measurements exceeding 1000 copies/mL [15].

According to Edet et al. [16], sustaining a VL level of 50 copies/mL is linked to the attainment of enduring clinical advantages. Conversely, the CD4 cell response to ART exhibits greater variability. Following ART initiation, there is a rapid increase in the CD4 cell count during the initial month, ranging from 75 to 100 cells/mL, with a subsequent more gradual response thereafter [17]. Notwithstanding viral suppression, certain patients exhibit a lack of increase in CD4 cell count, causing an “immunovirologic discordant” CD4 cell response. Multiple factors have been proposed as potential causes of this phenomenon. Insufficient thymic activity and persistent viral replication, despite effective viral suppression, have been postulated as potential explanatory factors [18].

Numerous factors have demonstrated an association with an elevated risk of virologic failure. Nonadherence to ART and specific age groups have been identified as contributing factors [19–21]. In addition, a higher VL at the initiation of treatment is linked to an increased incidence of viral failure [22]. Conversely, a higher baseline CD4 cell count, lower baseline VL, and more favorable clinical stage at treatment initiation are associated with achieving viral suppression at an earlier stage [19].

The objective of this study was to evaluate the impact of HAART outcomes among adult individuals residing in the high-incidence region of KwaZulu-Natal Province, South Africa. The key indicators under investigation include viral suppression, immune recovery, and mortality rate. In addition, the study aims to identify and analyze the factors associated with these specific outcomes.

## 2. Materials and Methods

### 2.1. Study Design and Setting

In this retrospective longitudinal cohort study, people living with HIV and AIDS (PLWHA) adult patients were recruited from the Antiretroviral (ARV) clinic at King Edward VIII Hospital in Durban, located in the KwaZulu-Natal Province of South Africa. The inclusion criteria involved patients who received care and initiated ART at the hospital, specifically targeting those who started ART in or after 2017. All relevant case files meeting these criteria were considered, and the study period was from January 2017 to December 2021. The study site provides daily care for PLWHA and serves as a primary point of contact for approximately 2300 PLWHA, accommodating an average of 4000 visits annually. Among these patients, 1301 are currently receiving ART from the study site. Furthermore, an average of 10 patients is initiated on ART at the clinic each month.

### 2.2. Study Participants

Adult patients with HIV infection were identified by reviewing the medical records from King Edward VIII Hospital. The inclusion criteria encompassed case files of patients who started ART in 2017 or later. Moreover, only case files of patients aged 18 years or older at the time of ART initiation were considered. Exclusions were made for medical case notes that were unaccounted for due to reasons such as loss or misfiling, incomplete information, or patients being transferred to another level of care. In addition, participants who had been receiving ART for less than 6 months were excluded from the study, as this timeframe was deemed the minimum requirement for assessing treatment response [15, 23].

### 2.3. Analyzing Variables for Treatment Outcomes

Within the subsequent course of action, adult individuals infected with HIV, who are under the care and started ART at the ARV clinic located within the premises of King Edward VIII Hospital, partake in biannual appointments. During these visits, healthcare practitioners evaluate and document a range of variables related to treatment efficacy such as VL and CD4 cell count. Furthermore, instances of mortality are diligently recorded.

The exposure variable under scrutiny was ART, whereas the principal outcome variable of interest revolved around the achievement of viral suppression at 6 and 12 months after the initiation of ART. Additional outcomes of significance included alterations in CD4 cell count and mortality occurrences during the follow-up period. Virologic suppression was explicitly defined as the attainment of HIV VLs below 50 copies/mL after 6 months of ART administration [15, 24].

To account for potential confounding factors, patient age, sex, marital status, presence of opportunistic infections, harmful behaviors, and WHO staging were meticulously recorded and subsequently incorporated into the analysis.

The first-line antiretroviral regimen consisted of a fixed-dose combination (FDC) of tenofovir, lamivudine, or emtricitabine, and dolutegravir. Second-line combination ART (cART) comprised of zidovudine or tenofovir, lamivudine, and ritonavir-boosted lopinavir or dolutegravir. The third-line regimen was usually given to the patients who had a treatment failure decided by a review committee [25].

### 2.4. Statistical Analysis

The study dataset was obtained from physical patient records stored in paper folders. Subsequently, Microsoft Excel 2021 was employed to transform the data into a format suitable for analysis using STATA software Version 18.0. The analysis encompassed the use of both descriptive and inferential statistical methods to delineate the demographic and clinical variables, at baseline, 6, and 12 months following the initiation of ART. The dataset containing sociodemographic and treatment outcome parameters was summarized. Continuous variables are depicted in terms of their means, whereas categorical variables are represented as percentages or proportions. The chi-square test was employed to assess the equality of categorical variables. To investigate the factors linked with virologic, immunological, and clinical responses, a comprehensive analysis involving both univariate and multivariate logistic regression techniques was conducted. The initial step encompassed univariate analysis, in which crude odd ratios were computed to determine the associations between variables and immunologic, virologic, and clinical responses. The variables under scrutiny included age, gender, marital status, presence of opportunistic infections, WHO staging, and injurious behaviors. The variables in the univariate analysis were integrated into the multivariate models, and their corresponding adjusted odd ratios were computed. The estimates were accompanied by 95% confidence intervals (CIs), and statistical significance was deemed to be present at a *p* value threshold of 0.05.

### 2.5. Ethical Considerations

This study received ethics approval from the University of KwaZulu-Natal Biomedical Research Ethics Committee under the reference number: BREC/00004657/2022. The management of the King Edward VIII Hospital, South Africa, permitted the study after approval from the KwaZulu-Natal Department of Health.

## 3. Results

### 3.1. Sociodemographic and Clinical Characteristics

After excluding the patients who had been on treatment for less than 6 months, and those demonstrating no observable results post the 6-month threshold, an examination of the clinical records of 707 patients was undertaken and subsequently used for comprehensive analysis. The sociodemographic and clinical characteristics of the patients are defined and presented in Table 1. Among the cohort of HIV-positive adult individuals under investigation, consisting of 707 patients over the specified study duration, 304 (43%) were male and, 403 (57%) were female. Regarding age distribution, 398 (56.3%) fell within the 18–39 years range, with 309 (43.7%) encompassing ages exceeding 40 years. In terms of marital status, the demographic profile revealed that 447 (63.23%) individuals were unmarried, 125 (17.68%) were married, 119 (16.8%) were widowed, and 16 (2.26%) were divorced. In addition, a subset of 127 patients (17.96%) exhibited detrimental behaviors, notably substance use.

Among this cohort of 707 adult HIV patients, 157 (22.21%) presented with opportunistic diseases at the ART initially. Moreover, 160 (22.63%) deaths were reported during the research period. According to the WHO stage classification, 440 patients (62.24%) were initially categorized within the WHO Stage I classification, 119 (16.83%) were in Stage II, 79 (11.17%) were in Stage III, and 69 (9.76%) were in Stage IV (Table 1).

### 3.2. Virologic Response

Among the initial cohort of 707 patients, distinct distributions of VL were evident: 171 individuals (24.19%) exhibited a VL ranging from 1001 to 100,000 copies/mL, 187 individuals (26.45%) displayed a VL spanning from 201 to 1000 copies/mL, 229 individuals (32.39%) showed a VL within the range of 50–200 copies/mL, and 120 individuals (16.97%) had a VL of less than 50 copies/mL. Upon the elapse of 6 months of ART, favorable suppression of VL was observed in 318 (44.98%) patients. Subsequently, at the 12-month mark, viral suppression was evident in 474 patients, constituting 67.04% of the total study population. Notably, the prevalence of viral suppression demonstrated a consistent upward trajectory with prolonged ART intervention. A subset of 318 (44.98%) patients achieved the benchmark of two consecutive instances of suppressed VLs. In addition, a distinct subgroup comprising 53 patients (7.50%) showcased a pattern of two consecutive VL readings up to 1000 copies/mL, observed 6 months apart.

### 3.3. Immunological Response

A consistent increase was observed in the proportion of patients exhibiting CD4 cell counts exceeding 500 cells per mm^3^, with the baseline average of 22.91% (*n* = 162) registering an increase to 48.94% (*n* = 346) in the 6^th^ month and further escalating to 79.49% (*n* = 562) by the 12^th^ month following the commencement of ART. Concurrently, the percentage of adult patients characterized by markedly diminished CD4 cell counts, specifically falling below 200, underwent a notable decline from 28.57% (*n* = 202) at baseline to 11.90% (*n* = 84) at the termination of the 6-month interval of treatment, ultimately diminishing further to 9.80% (*n* = 69) at the culmination of the 12 months.

### 3.4. Mortality Rate

Among the initial cohort of 707 patients, 160 deaths were recorded, constituting approximately 22.63% of the observed cases within the designated research duration. In the following years, 93 deaths (12.7%) were observed in the first year, 31 (4.2%) in the second year, and 15 (2.12%), 13 (1.98%), and 8 (1.55%), respectively.

### 3.5. Factors Associated With Immunologic and Virologic Responses and Mortality Among Individuals on HAART in KwaZulu-Natal, South Africa

As shown in Table 2, univariate analysis revealed a statistically significant association between viral suppression and baseline VL among the variables studied (VL: 50–200 copies/mL and VL: < 50 copies/mL). Other factors significantly associated with viral suppression are age (40 years and above) and no presence of harmful behaviors. These results were consistent with those of the subsequent multivariate logistic regression analysis. Specifically, the cohort with VL: < 50 copies/mL exhibited substantially higher odds (adjusted odds ratio [AOR] of 13.66, 95% CI: 2.46–75.63; *p* ≤ 0.01) of achieving a sustained viral suppression of at least two consecutive measurements. Following this, the group with VL: 50–200 copies/mL demonstrated an AOR of 5.28 (95% CI: 2.06–13.53; *p* ≤ 0.01). In addition, the age group of 40 years and above had an AOR of 1.21 (95% CI: 0.62–2.36; *p*=0.04). The absence of harmful behaviors had an AOR of 1.05 (95% CI: 0.48–2.26; *p*=0.03).

The factors associated with a suitable CD4 cell count response, as shown in Table 3, were gender, age, and harmful behaviors. Other factors associated with immunological responses were WHO staging and CD4 cell count at baseline. Multivariate analysis showed that the above factors had higher odds of attaining an adequate immunological response. The age group of 18–39 years and WHO Staging I had an AOR of 10.83 (95% CI: 3.04–38.44; *p* ≤ 0.01). The absence of harmful behaviors had an AOR of 2.19 (95% CI: 1.15–4.17; *p*=0.01). The female gender had an AOR of 1.23 (95% CI: 0.73–2.08; *p*=0.04). The group CD4: 200–500 cells per mm^3^ had an AOR of 5.11 (95% CI: 2.69–9.70; *p* ≤ 0.01). In addition, the CD4 group > 500 cells per mm^3^ had an AOR of 5.55 (95% CI: 1.78–17.28; *p* ≤ 0.01).

The factors associated with mortality, as shown in Table 4, were gender, age, marital status, and harmful behaviors. The other factors associated with mortality were VL at baseline, CD4 cell count at baseline, and opportunistic diseases.

Multivariate analysis showed that the above factors had higher odds of being associated with mortality. The gender male, age group of 18–39 years, being unmarried, presence of harmful behaviors, VL at baseline: 10,001–100.000 copies/mL, the group of CD4: < 200 mm^3,^ and presence of opportunistic diseases had an AOR of 4.62 (95% CI: 1.49–14.34; *p* ≤ 0.01).

## 4. Discussion

This study reviewed immunologic and virologic responses and mortality rates and identified the factors associated with these variables in PLWHA who are on HAART residing in the high-incidence region of KwaZulu-Natal Province, South Africa, over a 12-month after ART initiation.

The study identified that more females were initiated on HAART than males and most people in the cohort were aged 18–39 years and unmarried. A study conducted by Johnson et al. [26] in South Africa, acknowledged that females appeared to have more acceptance to HAART than males. Pregnancy and breastfeeding serve as pivotal moments for women to undergo HIV testing and start ART. Conversely, there is currently a lack of similar entry points designed for men and most children. Govender et al. [27] revealed that younger people, especially those who are unmarried, have a higher probability of being HIV positive because they have a higher number of lifetime sexual partners.

The findings from this study delineated that the percentage of individuals achieving viral suppression exhibited a consistent rise over time, in accordance with the study conducted by Edet A et al. [16].

Our study revealed that 318 (44.98%) and 474 (67.04%) of the total patients in our cohort were virologically suppressed at 6 and 12 months, respectively. A total of 318 (44.98%) patients achieved the benchmark of two consecutive instances of suppressed VLs. This study's finding corroborates with the study conducted by Crepaz et al. [28] and Ikpe et al. [29], who confirmed in their studies that the prevalence of viral suppression has demonstrated a consistent upward trajectory with prolonged ART intervention.

Despite this overall achievement in treatment outcomes, our study finding identified that the study participants fell short of meeting the 90-90-90 goals established by the United Nations Program on HIV/AIDS (UNAIDS), which aimed for 90% of HIV-infected patients on ART to attain VL suppression by 2020 [30]. More recently, this objective has been elevated to 95% to be achieved by 2030 [31]. Our findings were supported by the research conducted by Hermans et al. [32], on investigating virological suppression and clinical interventions in response to viremia within the South African HIV treatment program, which revealed a viral suppression rate of 73.1% below 50 copies/mL at 12 months and 77.5% at 72 months of ART administration. However, it must be stated that this low percentage of patients maintaining consistent viral suppression poses potential challenges to using viral suppression as a prevention strategy.

The initial rapid achievement of viral suppression is crucial in the sustained success of treatment among all individuals on ART. Rapid suppression is instrumental in fostering immune recovery and frequently serves as a deterrent to the emergence of viral resistance [33]. The continual presence of HIV viremia among ART recipients significantly accelerates the onset of viral resistance, consequently culminating in treatment failure [34]. Attaining viral suppression fosters immune reconstitution and contributes to tangible clinical improvement.

Despite this achievement, some patients did not achieve viral suppression, even after 6–12 months of ART. We believe that these patients may have inadequate adherence to medication schedules or irregular drug intake, which could lead to incomplete suppression of the virus. This incomplete suppression could create an environment in which the virus could mutate and develop resistance to the drugs being used. HIV resistance testing is advised for these patients.

In our study, an increase in immune recovery was observed in patients showing CD4 cell counts above 500 mm^3^, with a baseline average of 22.91%–48.94% in the 6^th^ month and further rising to 79.49% by the 12^th^ month following the initiation of ART. A similar pattern was observed in a South African study conducted by Edet et al. [16] in Limpopo Province, which showed an increase in immune recovery in terms of mean CD4 cell count of 227 mm^3^ at baseline to 372 mm^3^ at 6 months and 419 mm^3^ at 12 months [16].

However, in our cohort, some patients did not achieve immune recovery even after 12 months of treatment. In general, the increase in CD4 cell count during ART can continue even during the second year and over time [35].

Throughout our study, 160 patients died from HIV-related causes, representing a mortality rate of 22.63%. In the first year, 93 deaths were documented, constituting a mortality rate of 12.7%. Subsequently, 31 deaths, equivalent to 4.2%, occurred during the second year. In the ensuing years, the observed mortality rates were 2.12%, 1.98%, and 1.55%, corresponding to 15, 13, and 8 deaths, respectively. This result could be due to the delay in HIV diagnosis for these patients, as reported by Bakewell et al. [36] in their study estimating the risk of mortality attributable to recent late HIV diagnosis. It has been shown that a delay in HIV diagnosis is associated with a high mortality rate. In their study, they found a mortality rate of 38% for patients who did have a late HIV diagnosis versus 22% for those without a late diagnosis. However, our finding aligns with the investigation conducted in Kenya by Silverman et al. [37], where it was documented that the mortality incidence rate within the first year of initiating ART ranged between 7.44% and 12.78%.

The factors significantly associated with at least two consecutive suppressed VLs were VL at baseline and no presence of harmful behaviors (OR = 1.05, *p*=0.03), patients with VL: < 50 copies/mL (OR = 13.66, *p* ≤ 0.01) and the group with VL: 50–200 copies/mL (OR = 5.28, *p* ≤ 0.01), and females (OR = 1.46, *p*=0.02) who were more likely to achieve viral suppression at 6 months. The age group of 40 years and above (OR = 1.21, *p*=0.04) also had a better virologic response.

The studies conducted in Africa by Bahemana et al. [38] evaluating the impact of age on CD4 recovery and viral suppression in HIV patients, and Wakooko et al. [39] in Bulambuli District, Uganda, assessing VL suppression and associated factors in HIV patients, found that age group was not associated with VL suppression. However, our study found that old age was associated with viral suppression. The extant literature confirms that the older age group demonstrates the capacity to attain viral suppression, as they exhibit a proclivity for consistently adhering to their ART regimen independently. This ability indicates a heightened level of self-efficacy and self-competency in the context of ART adherence [40]. Some studies have shown that harmful behaviors of substance use are associated with not being virally suppressed during follow-up [41, 42], as shown in our research. In addition, our study found that a better baseline VL was associated with viral suppression, as mentioned by the research performed by Opoku et al. in Ghana [34]. Inversely, few studies found that the young age group of adults was associated with favorable immune recovery. In this active age, patients have excellent thymus functions, as indicated in the study performed by Silva et al. [43]. In addition, these patients can work and self-manage their conditions, including food and medication adherence, which can lead to a favorable immune recovery [44].

In this study, we identified some factors associated with favorable immunological responses. These factors included female gender (OR = 1.23, *p*=0.04), age group of 18–39 years (OR = 10.83, *p* ≤ 0.01), no presence of harmful behaviors (OR = 2.19, *p*=0.01), early WHO clinical staging at baseline (OR = 10.83, *p* ≤ 0.01), and higher baseline CD4 counts (OR = 5.55, *p* ≤ 0.01). This finding aligns with the research conducted by Gunda et al. [45], which documented that the factors associated with poor immune recovery were male gender, older age, low baseline CD4 counts, and advanced WHO clinical stage [34].

Several studies revealed that male patients have a poor and slower immune recovery compared to women [46]. Many factors are linked to this phenomenon, including the hormonal regulatory effect on CD4 numbers and inflammatory responses, genetic factors, higher rates of comorbid conditions in men, and behavioral and health-seeking patterns. In this context, it has been demonstrated that females exhibit relatively elevated CD4 levels compared to males, which are estrogen-regulated. In the presence of suppressive HAART, this inherent homeostatic regulation of CD4 cell count may consequently result in a greater increase in CD4 levels among females than males [47]. Females have two X chromosomes, and many immune-related genes are located on the X chromosome. This genetic advantage may contribute to a more effective immune response in women [48]. Men often have higher rates of comorbid conditions such as cardiovascular diseases or substance abuse, which can negatively affect immune function and slow recovery [49]. Studies have shown that men often seek healthcare later than women, including in the context of HIV. This delay in treatment initiation means that men may start ART at a more advanced stage of immunosuppression, leading to slower immune recovery [50].

Men may also have lower adherence to ART compared to women, which can negatively impact immune recovery.

A higher baseline CD4 count was another factor associated with favorable immune recovery. Kelley et al. [51]'s investigation revealed that 93% of patients diagnosed with HIV, and initiated on ART with baseline CD4 counts ranging from 200 to 350 cells/mm^3^, achieved subsequent CD4 levels within the range of 350–500 cells/mm^3^ after 4 years [51]. Notably, patients initiating ART with a baseline CD4 count below 200 cells/mm^3^ exhibited compromised immune recovery, as evidenced by the study conducted by Fiseha et al., in which only 30% of patients initiating ART with CD4 counts below 200 cells/mm^3^ achieved CD4 counts within the range of 350–500 cells/mm^3^ after 12 months [52].

It was also indicated that patients with baseline WHO clinical Stage 1 were more likely to receive a favorable immune recovery on ART than those with baseline WHO clinical Stages 3 and 4.

The study performed by Ilovi et al. [53] confirmed this finding. It indicated that patients in the early HIV stage had suitable immune recovery compared to those in the advanced stage of the disease [53].

Our study found that no harmful behaviors were associated with favorable immune recovery, as shown by Ladak et al., who indicated that substance abuse fosters poor immune recovery in HIV patients [54].

In our study, some factors were associated with mortality. Those factors included male gender, age group of 18–39 years, marital status unmarried, presence of harmful behaviors, higher VL at baseline, lower CD4 at baseline, and presence of opportunistic diseases, with OR = 4.62 and *p* ≤ 0.01.

As indicated in the study performed by Leung et al. [55], our study also found that mortality was associated with male gender and being unmarried.

The reason is that certain high-risk sexual behaviors, including inconsistent condom usage, engagement in multiple sexual partnerships, and drug or alcohol abuse, significantly elevate the susceptibility of males and unmarried to HIV infection, leading to high mortality rates [56, 57].

The second explanation for their high mortality is that men exhibit adverse effects associated with ART and demonstrate lower adherence to treatment protocols than women [58]. Consequently, concerted efforts should be directed toward promoting greater engagement of HIV-infected males in ART initiatives, emphasizing the importance of early intervention for optimal outcomes.

The third reason is related to the high rate of unemployment among working-aged men in KwaZulu-Natal Province [59]. This situation fosters social vulnerability issues for young men, leading to high mortality rates.

In this study, mortality was also associated with the younger age group of adults, as revealed by the study by Amour et al. [60]. This observation aligns with the potential hypothesis that youth may delay seeking care because of the stigma and fear associated with HIV and AIDS and/or encounter barriers to accessing testing services.

Our study identified that low baseline CD4 and high baseline VL levels were associated with mortality. This analysis was consistent with the study conducted by Bisson et al. [61], which indicated that high baseline VL and low baseline CD4 were increasing the risk of mortality. The literature suggests a robust correlation between early mortality and the extent of immunodeficiency at the initiation of ART [62], as is the case in our study. Therefore, strategies to reduce mortality must include earlier HIV diagnosis and enhanced longitudinal care preceding ART initiation.

Our study highlighted that mortality is associated with harmful behaviors. The study by Fontela et al. [63] in Spain showed that HIV patients with a history of injecting drug use had a high risk of mortality. In addition, Sarah et al. [64] indicated that alcohol use increased the risk of mortality in individuals living with HIV.

The presence of opportunistic infections was found to be associated with mortality, as highlighted in the study by Meng et al. [65], which reported a higher in-hospital mortality rate of 11.2% among HIV/AIDS patients with opportunistic infections, compared to an overall in-hospital mortality rate of 9.0%.

### 4.1. Study Limitations

Our study had a few limitations: our investigation was single-site clinic-based, so results may not be generalizable to the overall population. As a retrospective study, the data were collected from patients' files, these are mostly incomplete; some of the critical variables were missing, such as CD4 counts and VL measurements for some patients. Therefore, this sample had to be excluded from the study. Despite the limitations delineated above, this study has several strengths, including a relatively large sample size study that evaluated the virological suppression, immune recovery, and mortality rates in patients initiated on HAART.

## 5. Conclusion

The evaluation of treatment outcomes among individuals on HAART in KwaZulu-Natal, South Africa, underscores the transformative impact of HAART on HIV management in a region with one of the highest HIV prevalence rates globally. Our study demonstrates that HAART is effective in achieving virological suppression and immunological recovery, substantially reducing the incidence of opportunistic infections and overall mortality. In addition, the proportion of suitable immunological responses for individuals on ART increased over time.

Despite these positive outcomes, several challenges persist. Some patients do not achieve optimal virological suppression, and the percentage of HIV-infected patients attaining viral suppression at 6 and 12 months was below the 90% target established by the UNAIDS.

This study will contribute to the existing body of knowledge by highlighting the gaps in understanding treatment outcomes among adults on HAART in the high-incidence setting of KwaZulu-Natal Province. The results will have implications for healthcare practitioners, policymakers, and researchers, offering insights into potential strategies for improving the management and outcomes of HAART in this region.

## Figures and Tables

**Table 1:** Sociodemographic and clinical characteristics of HIV-infected adults on highly active antiretroviral therapy in KwaZulu-Natal, South Africa.

Characteristics	Total (*N* = 707)	Percentage (%)
*Gender*		
Male	304	43.00
Female	403	57.00

*Age at ART start (years)*		
18–39 years	398	56.30
40 and above	309	43.70

*Marital status*		
Unmarried	447	63.23
Married	125	17.68
Widowed	119	16.83
Divorced	16	2.26

*Harmful behaviors (substance use)*		
Yes	127	17.96
No	580	82.04

*CD4 account at the first visit*		
CD4 < 200 mm^3^	202	28.57
CD4: 200–500 mm^3^	343	48.52
CD4 > 500 mm^3^	162	22.91

*CD4 account after 6 months*		
CD4 < 500 mm^3^	361	51.06
CD4 > 500 mm^3^	346	48.94

*CD4 account after 12 months*		
CD4 < 500 mm^3^	145	20.51
CD4 > 500 mm^3^	562	79.49

*Viral load at the first visit*		
VL: 1001–100,000 copies/mL	171	24.19
VL: 201–1000 copies/mL	187	26.45
VL: 50–200 copies/mL	229	32.39
VL: Less than 50 copies/mL	120	16.97

*Viral load after 6 months*		
VL: More than 50 copies/mL	389	55.02
VL: Less than 50 copies/mL	318	44.98

*Viral load after 12 months*		
VL: More than 50 copies/mL	233	32.96
VL: Less than 50 copies/mL	474	67.04

*WHO stage at ART initiation*		
I	440	62.24
II	119	16.83
III	79	11.17
IV	69	9.76

*Opportunistic diseases at the first visit*		
Yes	157	22.21
No	550	77.79

*Dead under research period*		
Yes	160	22.63
No	547	77.37

**Table 2:** Variables associated with viral suppression (at least two consecutive suppressed viral loads).

Characteristic	Unadjusted OR (95% CI)	*p *value	Adjusted OR (95% CI)	*p* value
*Gender*				
Male	1.78 (1.41–2.26)	≤ 0.01	3.80 (0.97–14.84)	0.05
Female	1.27 (0.92–1.74)	0.13	1.46 (0.81–2.64)	0.20

*Age (years)*				
18–39	1.72 (1.40–2.11)	≤ 0.01	3.80 (0.97–14.84)	0.05
40 and above	1.50 (1.08–2.07)	0.01	1.21 (0.62–2.36)	0.04

*Marital status*				
Unmarried	1.82 (1.50–2.22)	≤ 0.01	3.80 (0.97–14.84)	0.05
Married	1.30 (0.84–1.99)	0.23	1.53 (0.66–3.54)	0.31
Widowed	1.62 (1.02–2.56)	0.03	1.41 (0.60–3.32)	0.42
Divorced	0.70 (0.25–1.92)	0.49	0.90 (0.13–6.60)	0.95

*Harmful behaviors*				
Yes	0.92 (0.65–1.30)	0.65	3.80 (0.97–14.84)	0.05
No	2.69 (1.82–3.99)	≤ 0.01	1.05 (0.48–2.26)	0.03

*WHO staging at ART initiation*				
I	22.15 (13.99–35.08)	13.99	3.80 (0.97–14.84)	0.05
II	0.03 (0.01–0.05)	0.01	0.06 (0.02–0.12)	≤ 0.01
III	0.00 (0.00–0.01)	≤ 0.01	0.00 (0.00–0.02)	≤ 0.01
IV	0.00 (0.00–0.00)	≤ 0.01	0.00 (0.00–0.01)	≤ 0.01

*VL at baseline (copies/mL)*				
VL: 1001–100,000	0.22 (0.15–0.32)	≤ 0.01	3.80 (0.97–14.84)	0.05
VL: 201–1000	7.72 (4.73–12.60)	≤ 0.01	2.65 (1.23–5.68)	0.07
VL: 50–200	47.19 (25.86–86.12)	≤ 0.01	5.28 (2.06–13.53)	≤ 0.01
VL: < 50	176.12 (52.50–590.78)	≤ 0.01	13.66 (2.46–75.63)	≤ 0.01

*CD4 baseline (mm* ^3^)				
CD4 < 200	0.44 (0.32–0.59)	≤ 0.01	3.80 (0.97–14.84)	0.05
CD4: 200–500	7.07 (4.79–10.42)	≤ 0.01	0.55 (0.26–1.15)	0.11
CD4: > 500	38.38 (18.39–80.11)	≤ 0.01	0.68 (0.21–2.16)	0.51

*Opportunistic diseases at baseline*				
Yes	0.09 (0.05–0.16)	≤ 0.01	3.80 (0.97–14.84)	0.05
No	52.90 (29.21–95.82)	≤ 0.01	1.34 (0.56–3.24)	0.50

**Table 3:** Variables associated with favorable immunological responses.

Characteristic	Unadjusted OR (95% CI)	*p* value	Adjusted OR (95% CI)	*p* value
*Gender*				
Male	3.34 (2.55–4.36)	≤ 0.01	10.83 (3.04–38.44)	≤ 0.01
Female	1.28 (0.89–1.85)	0.01	1.23 (0.73–2.08)	0.04

*Age (years)*				
18–39	3.47 (2.74–4.39)	≤ 0.01	10.83 (3.04–38.44)	≤ 0.01
40 and above	1.27 (0.87–1.84)	0.20	0.71 (0.39–1.29)	0.27

*Marital status*				
Unmarried	3.56 (2.84–4.45)	≤ 0.01	10.83 (3.04–38.44)	≤ 0.01
Married	0.92 (0.5–1.49)	0.76	0.78 (0.38–1.61)	0.51
Widowed	2.10 (1.15–3.84)	0.01	2.11 (0.94–4.73)	0.06
Divorced	0.61 (0.20–1.82)	0.38	0.65 (0.13–3.06)	0.59

*Harmful behaviors*				
Yes	1.59 (1.11–2.27)	0.01	10.83 (3.04–38.44)	≤ 0.01
No	3.12 (2.05–4.75)	≤ 0.01	2.19 (1.15–4.17)	0.01

*WHO staging at ART initiation*				
I	23.44 (14.62–37.57)	≤ 0.01	10.83 (3.04–38.44)	≤ 0.01
II	0.13 (0.07–0.24)	≤ 0.01	0.13 (0.05–0.30)	≤ 0.01
III	0.03 (0.01–0.05)	≤ 0.01	0.02 (0.00–0.83)	≤ 0.01
IV	0.01 (0.01–0.02)	≤ 0.01	0.10 (0.00–0.03)	≤ 0.01

*VL at baseline (copies/mL)*				
VL: 1001–100,000	0.96 (0.71–1.30)	0.81	10.83 (3.04–38.44)	≤ 0.01
VL: 201–1000	4.66 (2.89–7.51)	≤ 0.01	0.65 (0.32–1.33)	0.24
VL: 50–200	10.82 (6.25–18.72)	≤ 0.01	0.26 (0.09–0.68)	≤ 0.01
VL: < 50	23.82 (9.26–61.24)	≤ 0.01	0.29 (0.68–1.25)	0.09

*CD4 at baseline (mm* ^3^)				
CD4 < 200	0.88 (0.67–1.17)	0.39	10.83 (3.04–38.44)	≤ 0.01
CD4: 200–500	10.94 (6.93–17.28)	≤ 0.01	5.11 (2.69–9.70)	≤ 0.01
CD4: > 500	24.93 (11.13–55.84)	≤ 0.01	5.55 (1.78–17.28)	≤ 0.01

*Opportunistic diseases at baseline*				
Yes	0.72 (0.52–0.99)	0.04	10.83 (3.04–38.44)	≤ 0.01
No	12.40 (8.13–18.92)	≤ 0.01	0.79 (0.35–1.76)	0.59

**Table 4:** Factors associated with mortality.

Characteristic	Unadjusted OR (95% CI)	*p* value	Adjusted OR (95% CI)	*p* value
*Gender*				
Male	2.66 (2.06–3.42)	≤ 0.01	4.62 (1.49–14.34)	≤ 0.01
Female	1.54 (1.08–2.19)	0.016	1.41 (0.93–2.12)	0.10

*Age (years)*				
18–39	4.60 (3.56–5.95)	≤ 0.01	4.62 (1.49–14.34)	≤ 0.01
40 and above	0.52 (0.36–0.74)	≤ 0.01	0.55 (0.35–0.87)	0.01

*Marital status*				
Unmarried	4.73 (3.70–6.03)	≤ 0.01	4.62 (1.49–14.34)	≤ 0.01
Married	0.46 (0.29–0.73)	≤ 0.01	0.59 (0.34–1.01)	0.05
Widowed	0.43 (0.27–0.68)	≤ 0.01	0.41 (0.23–0.70)	≤ 0.01
Divorced	0.35 (0.12–0.99)	≤ 0.01	0.39 (0.11–1.36)	0.14

*Harmful behaviors*				
Yes	2.84 (1.91–4.23)	≤ 0.01	4.62 (1.49–14.34)	≤ 0.01
No	1.22 (0.78–1.90)	0.36	1.18 (0.68–2.05)	0.55

*WHO staging at ART initiation*				
I	5.37 (4.15–6.95)	≤ 0.01	4.62 (1.49–14.34)	≤ 0.01
II	0.44 (0.27–0.71)	≤ 0.01	0.73 (0.39–1.39)	0.35
III	0.40 (0.23–0.68)	≤ 0.01	0.82 (0.30–2.19)	0.69
IV	0.20 (0.11–0.34)	≤ 0.01	0.30 (0.10–0.91)	0.03

*VL at baseline (copies/mL)*				
VL: 10,001–100.000	2.48 (1.78–3.46)	≤ 0.01	4.62 (1.49–14.34)	≤ 0.01
VL: 201–1000	0.73 (0.47–1.15)	0.18	0.47 (0.25–0.90)	0.02
VL: 50–200	1.78 (1.11–2.86)	0.01	0.76 (0.35–1.64)	0.48
VL: < 50	9.23 (3.55–24.00)	≤ 0.01	1.85 (0.56–6.12)	0.31

*CD4 at baseline (mm* ^3^)				
CD4 < 200	2.81 (2.05–3.84)	≤ 0.01	4.62 (1.49–14.34)	≤ 0.01
CD4: 200–500	0.80 (0.54–1.18)	0.27	0.42 (0.24–0.73)	≤ 0.01
CD4 > 500	14.05 (4.96–39.77)	≤ 0.01	3.82 (1.16–12.53)	0.20

*Opportunistic diseases at baseline*				
Yes	1.27 (0.93–1.74)	0.01	4.62 (1.49–14.34)	≤ 0.01
No	3.84 (2.61–5.66)	≤ 0.01	2.98 (1.36–6.51)	0.31

## Data Availability

De-identified datasets used and/or analyzed during the current study are available from the corresponding author upon reasonable request.
